# Therapeutic efficacy of neural stem cells originating from umbilical cord-derived mesenchymal stem cells in diabetic retinopathy

**DOI:** 10.1038/s41598-017-00298-2

**Published:** 2017-03-24

**Authors:** Wei Zhang, Yuexin Wang, Jiahui Kong, Meng Dong, Hongtao Duan, Song Chen

**Affiliations:** Tianjin Eye Hospital, Tianjin Key Lab of Ophthalmology and Visual Science, Tianjin Eye Institute, Clinical College of Ophthalmology Tianjin Medical University, Tianjin, 300020 China

## Abstract

The aim of this study was to evaluate the effects of intravitreal injection of neural stem cells (NSCs) originating from human umbilical cord-derived mesenchymal stem cells (UC-MSCs) on neurodegeneration of diabetic retinopathy (DR) in rats. UC-MSCs were isolated and passaged, followed by induction to NSCs in neural differentiation medium. Four weeks following NSC transplantation, treatment attenuated retinal vascular dysfunction compared with non-treated rats, and BDNF and Thy-1 expression was significantly higher in the treated group than in the control group. Treatment of diabetic rats with NSCs prevented the decrease in BDNF levels caused by diabetes. The average leakage of Evans Blue (EB) dye in the treated group was significantly less than that in the control group. These morphological improvements were accompanied by a restoration of vision, as documented by F-ERG. NSCs originating from MSCs demonstrated a neuroprotective effect by increasing the number of surviving RGCs and significantly reducing the progression of DR. Thus, transplantation of NSCs could be a novel strategy for the treatment of neurodegeneration in DR.

## Introduction

Diabetic retinopathy (DR) has been considered a microcirculatory retinal disease caused by the deleterious metabolic effects of hyperglycemia^1^. The early pathogenesis of DR may be due to chronic degeneration of retinal nerve tissue, including reactive glial cell hyperplasia and neuronal apoptosis. Ongoing basic and clinical research is currently under way with the aim of curing neuroretinal disorders of DR^2^. Neuroretinal damage produces functional disorders, such as decreasing of contrast sensitivity, dark adaptation, and chromatic discrimination. In addition, neuroretinal degeneration activates multiple signaling and metabolic pathways that participate in microangiopathy and disruption of the blood-retinal barrier (BRB), a key factor in the pathogenesis of DR.

Mesenchymal stem cells (MSCs) are derived from the embryonic mesoderm during fetal development. MSCs are limited to bone marrow and several types of connective tissue in adults^3^. They are capable of cloning to form adherent fibroblastic cells that express the unique properties of the cell surface phenotype. Because the cells are highly expandable *ex vivo* and have an ability to differentiate along a variety of different cell lineages, they are considered resources for transplantation and stem cell-based therapy^4^. Human umbilical cord MSCs (UC-MSCs) have unique advantages and are attracting increasing attention in the clinic^5^. In addition, human umbilical cord appears to be more advantageous than other stem cell sources for cell procurement, storage, and transplantation^6^.

Therefore, MSCs are suitable for the development of cell-based treatments for various peripheral and central nerve injuries, and MSCs may act as a potential source for neural stem cell (NSC) therapies^7^. In our previous studies, we demonstrated the capacity of UC-MSCs to differentiate into NSCs *in vitro* by the combination of reverse transcription-polymerase chain reaction (RT-PCR) and immunofluorescence studies^8^. In the current study, we further investigated the effect of intravitreal transplantation of NSCs on long-term preservation of retinal function and delayed DR progression in rat.

## Results

### Cell culture

UC-MSCs were positive for the expression of CD73, CD90, and CD105 but weakly expressed CD34, CD45, CD11b, CD19, and HLA-D. NSCs were differentiated from UC-MSCs, and the expression of Nestin and NeuroD1 was detected in the NSCs as previously described^8^, suggesting that the molecular machinery for the neural function was activated in NSCs.

### Light microscopic analysis

**Figure 1:**
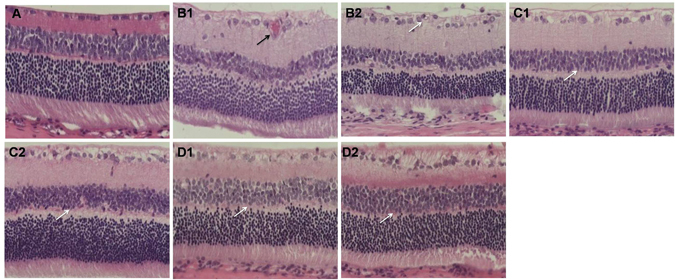
(**A**) Retinal HE staining in Group A. The retinal structure was continuous, and its capillary structure was normal. HE 200×. (**B1**–**2**) Retinal HE staining in Group B. The tissue layers of the retina were edematous, and the retina developed telangiectatic vessels (black arrow). Many ganglion cells developed vacuolar degeneration (white arrow) at 4 weeks (**B1**) and 8 weeks (**B2**). HE 200×. (**C** and **D**) Retinal HE staining in Groups C and D. Treatment with MSCs and NSCs attenuated the retinal vascular dysfunction, and the tissue layers of the retina were gradually arranged regularly (white arrow) at 4 weeks and 8 weeks. HE 200×.

In Group A, the structure of the retina was continuous with its normal capillary structure (Fig. 1A). The tissue layers of the retina developed telangiectatic vessels in the inner layer of retinas, and many ganglion cells developed vacuolar degeneration in the inner nuclear layer in Group B (Fig. 1B1–2). In Groups C and D, treatment with MSCs and NSCs attenuated the retinal vascular dysfunction, and the tissue layers of the retina were gradually arranged regularly (Fig. 1C1–2 and D1–2).

**Figure 2:**
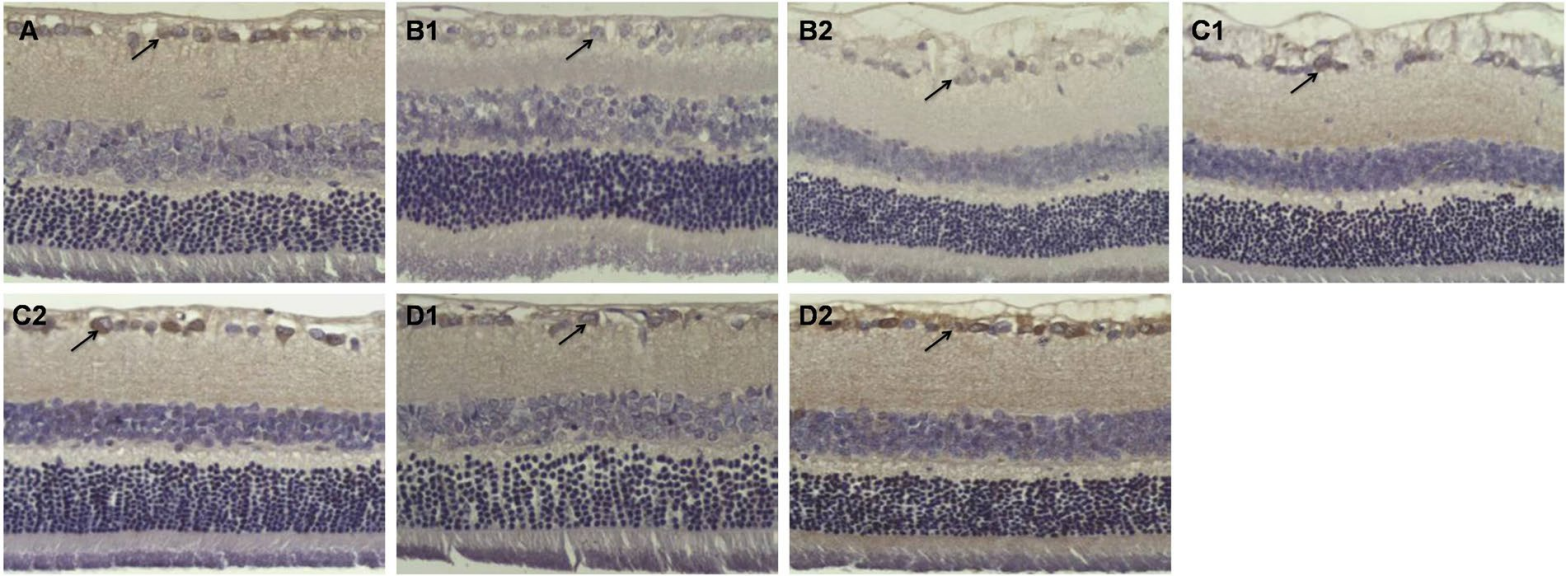
Expression of BDNF in the retina. (**A**) There were BDNF-positive cells in the ganglion cell layer and nerve fiber layer in Group A (arrow). Immunohistochemical staining 200×. (**B**) Four and eight weeks following cell transplantation, the number of BDNF-positive cells in the ganglion cell layer and external nuclear layer was decreased in Group B (arrow). Immunohistochemical staining 200×. (**C**,**D**) There was a marked increase in BDNF-positive cells in Groups C and D (arrow); the number increased specifically in Group D compared with Group C. Immunohistochemical staining 200×.

### BDNF and Thy-1 assay

**Figure 3:**
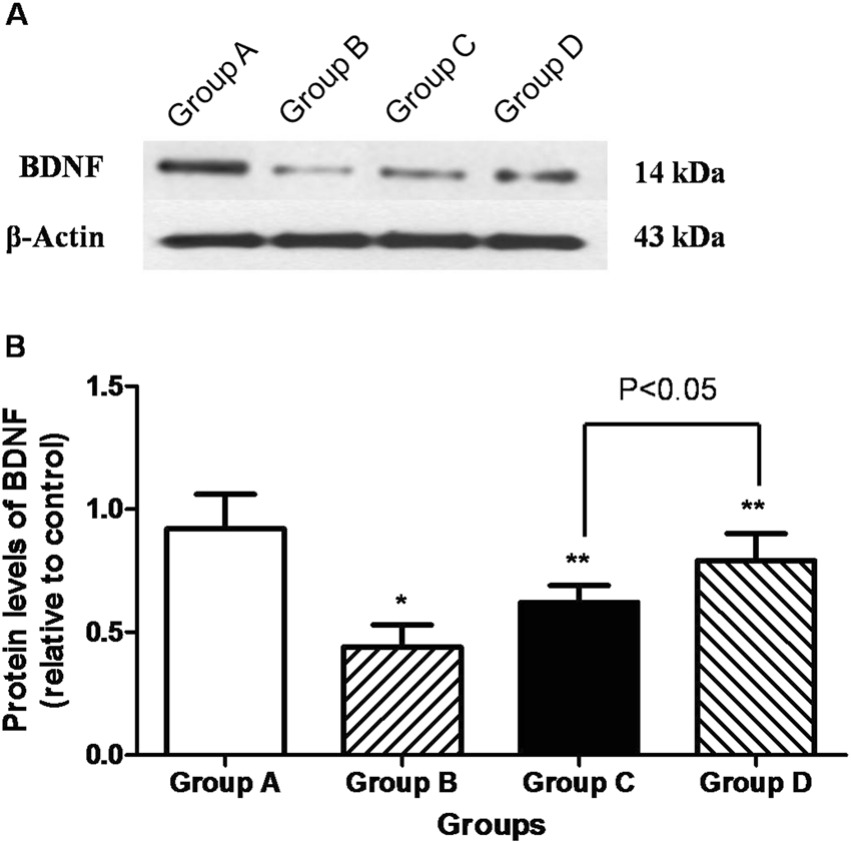
Western blotting analysis of BDNF in the retina. (**A**) Representative Western blot; (**B**) Western blot analysis. There were significant differences in the level of retinal BDNF protein between rats with DR (Group B: n = 6) and Group A (n = 6). In contrast, the level of BDNF protein increased in Groups C and D. Retinal BDNF protein levels in Group D were higher than that in Group C. *P < 0.05 vs. Group A; **p < 0.05 vs. Group B.

BDNF is involved in the survival and differentiation of RGCs in the retina and plays an important role in the formation and plasticity of synaptic connections. This can help to evaluate the neurotrophic degree of DR. We discovered that there were BDNF-positive cells in the ganglion cell layer and the nerve fiber layer in the control group (23.6 ± 2.8 n/HPF [high-power field]; Fig. 2A). Eight weeks after cell transplantation, the number of BDNF-positive cells in the ganglion cell layer and the external nuclear layer decreased in Group B (15.6 ± 2.6 n/HPF; Fig. 2B1). In contrast, the number of BDNF-positive cells in Group C (18.4 ± 3.2 n/HPF; Fig. 2C1) and Group D (20.4 ± 2.9 n/HPF; Fig. 2D1) increased compared with those in Group B (*p* < 0.05). Eight weeks following cell transplantation, the number of BDNF-positive cells dramatically decreased in Group B (9.6 ± 2.1 n/HPF; Fig. 2B2), and there was a marked increase in the number of BDNF-positive cells in Group C (16.6 ± 3.2 n/HPF; Fig. 2C2) and Group D (18.4 ± 2.8 n/HPF; Fig. 2D2); the number increased specifically in Group D compared with that in Group C (*p* < 0.05). The retinal BDNF protein in Group B (0.44 ± 0.09) was lower than that in Group A (0.92 ± 0.14) (*p* < 0.05). In contrast, the retinal BDNF protein increased in Group C (0.62 ± 0.07) and D (0.79 ± 0.11). In particular, the retinal BDNF protein in Group D was higher than that in Group C (*p* < 0.05) (Fig. 3).

**Figure 4:**
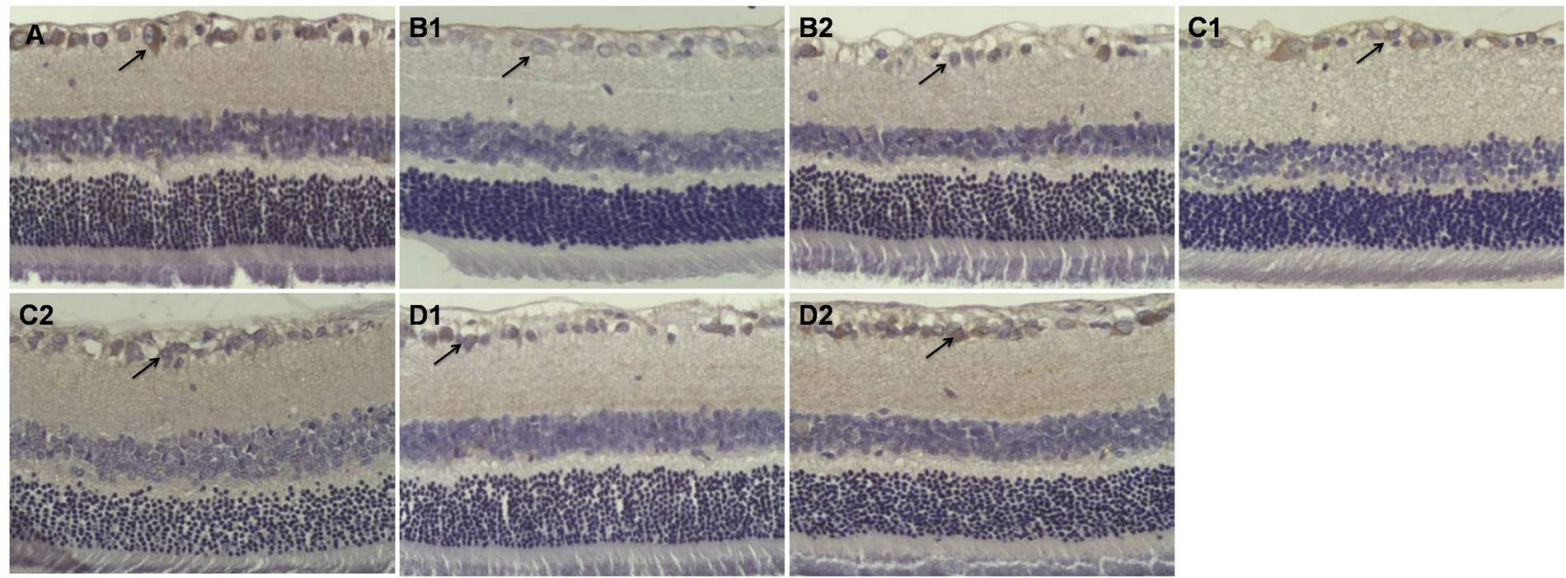
Thy-1-positive cells (the number of surviving RGCs) in the retina. (**A**) The number of Thy-1-positive cells was normal in Group A (25.2 ± 3.4 n/HPF) (arrow). Immunohistochemical staining 200×. (**B**) The number of Thy-1-positive cells was lower mainly in the ganglion cell layer in Group B (13.3 ± 1.9 n/HPF; *p* < 0.05) (arrow). Immunohistochemical staining 200×. (**C**,**D**) The number of Thy-1-positive cells was higher in Groups C and D than in Group B (*p* < 0.05) (arrow); the number was particularly higher in Group D (22.6 ± 2.8 n/HPF) than in Group C (18.1 ± 2.4 n/HPF; *p* < 0.05). Immunohistochemical staining 200×.

Immunohistochemical staining for Thy-1-positive cells (RGC marker expression indicating the number of surviving RGCs) in Group A was normal (25.2 ± 3.4 n/HPF; Fig. 4A). The number of Thy-1-positive cells gradually decreased mainly in the ganglion cell layer in Group B (13.3 ± 1.9 n/HPF; Fig. 4B) (*p* < 0.05). However, the number of Thy-1-positive cells increased in Group C (18.1 ± 2.4 n/HPF; Fig. 4C) and Group D (22.6 ± 2.8 n/HPF; Fig. 4D) compared with Group B; the number of Thy-1-positive cells increased specifically in Group D compared with Group C (*p* < 0.05).

### Measurement of BRB breakdown using EB dye

**Figure 5:**
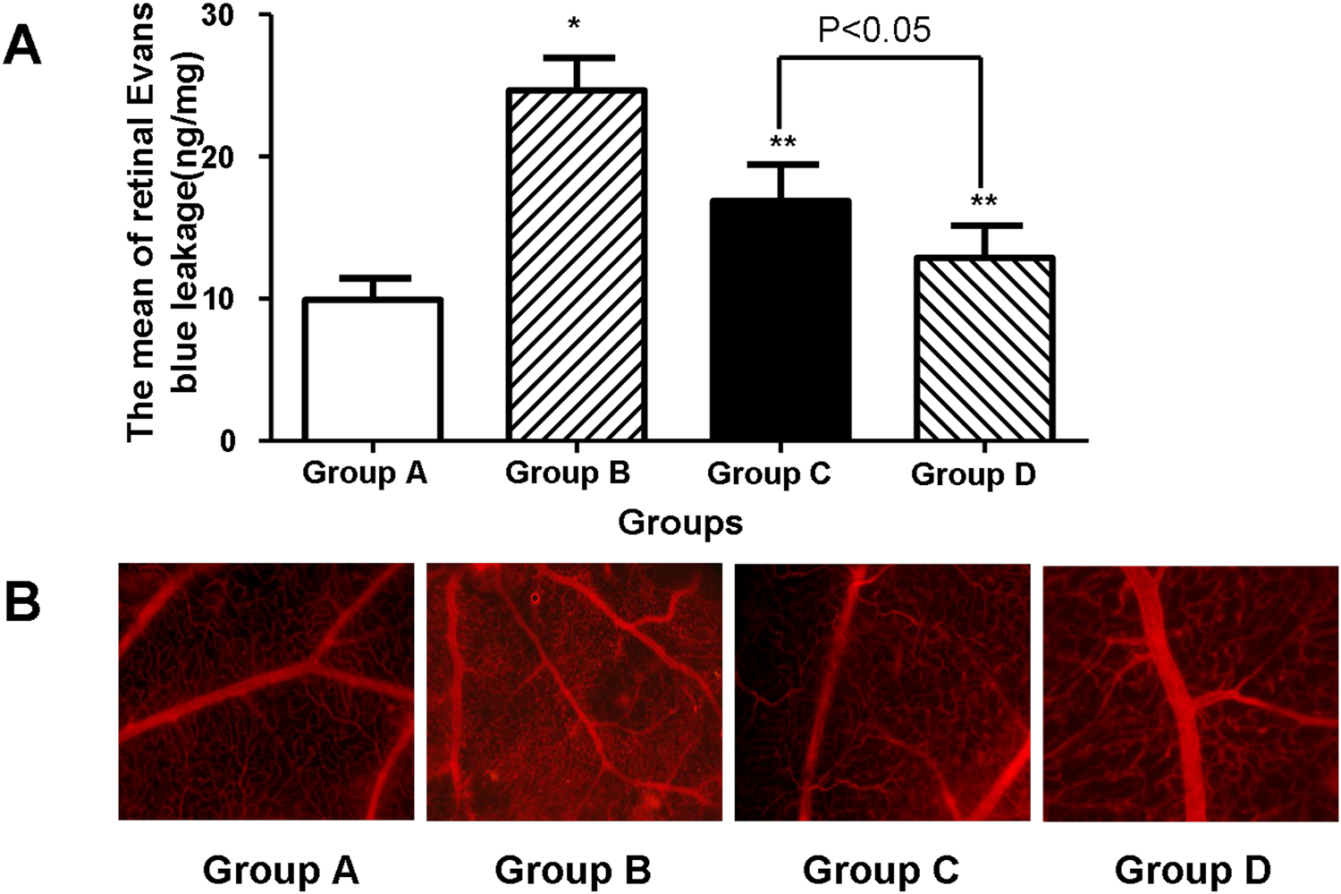
The protective effect of NSC on diabetic-induced BRB permeability. (**A**) Mean retinal EB dye leakage in the retinal blood vessels. There were significant differences in the retinal EB dye leakage between rats with DR (Group B: n = 6) and Group A (n = 6). In contrast, the leakage decreased in Groups C and D. Retinal EB dye leakage in Group D was lower than that in Group C. *P < 0.05 vs. Group A; **p < 0.05 vs. Group B. (**B**) Representative images showing EB fluorescence. In the retinas of Group A, EB fluorescence was limited to the blood vessels, whereas in Group B, the dye leaked out of the vessels to the retinal tissue (arrows). Treatment in Groups C and D prevented the leakage of EB. Magnification 400x.

Figure 5A shows the retinal blood vessel leakage of diabetic rats. The mean amount of retinal EB dye leakage in Group B (24.67 ± 2.26 ng/mg) was higher than that in Group A (9.91 ± 1.53 ng/mg) (*p* < 0.05). In contrast, retinal EB dye leakage decreased in Groups C (16.85 ± 2.58 ng/mg) and D (12.85 ± 2.27 ng/mg). Retinal EB dye leakage in Group D was lower than in Group C (*p* < 0.05). Retinal vessel leakage was also visualized with EB in retinal flat mounts (Fig. 5B). EB fluorescence was limited to the blood vessels in Group A. However, in the retina of Group B, EB leaked out of the vessels into the retinal tissue. Treatment in Groups C and D prevented this effect, corroborating the results obtained from the quantitative EB assay.

### Electroretinogram (ERG) recording

ERG testing enables the direct determination of photoreceptor (a-wave component) and second-order neuron (b-wave component) function. The function of cones and rods can be assessed by performing the test under light and dark adaptation, respectively. SD rats exhibit a prominent b-wave and a smaller a-wave, and both components decrease with age, as retinal degeneration progresses.

**Figure 6:**
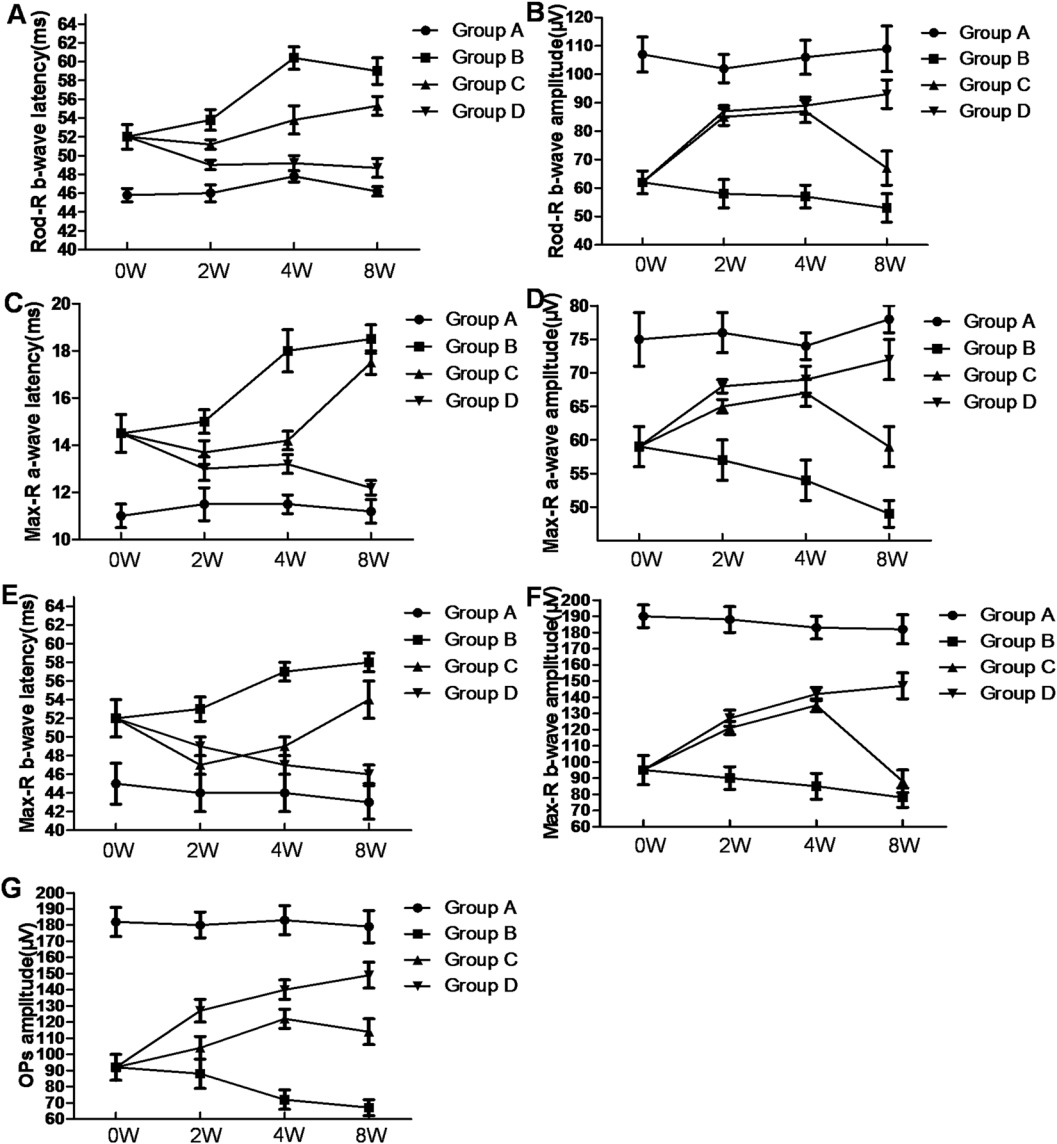
NSC transplantation enhanced b-wave amplitude and reduced the latency of ERG responses. (**A**,**B**) ERG responses (b-wave and a-wave) were significantly lower in Group B than in Group A. The mean maximal a-wave amplitudes were significantly higher in Groups C and D than in Group B at all time points between 2 and 8 weeks following cell transplantation. (**C**–**F**) Both Rod-R and Max-R b-wave recordings were significantly higher across light amplitudes in Groups C and D than in Group B at subsequent time points (2 to 8 weeks following transplantation). (**G**) The OP amplitudes were significantly higher in Groups C and D than in Group B at all time points between 2 and 8 weeks following cell transplantation. Data are presented as the mean ± SE.

Figure 6 shows NSC transplantation enhanced b-wave amplitude and reduced latency of ERG responses to light stimuli under dark and light adaptation in rats. The ERG responses (b-wave and a-wave) were significantly lower in Group B than in Group A. The mean maximal a-wave amplitudes were significantly higher in Groups C and D than in Group B at all time points between 2 and 8 weeks following cell transplantation (Fig. 6A,B) (F = 30.031, 21.119, *p* < 0.05). Both Rod-R and Max-R b-wave recordings were significantly higher across light amplitudes in Group C and D than in Group B at subsequent time points (2 to 8 weeks following transplantation) (Fig. 6C–F) (F = 12.562, 17.688, 27.548, 34.375, *p* < 0.05). The Oscillatorypotentials(OPs) amplitudes were significantly higher in Groups C and D than in Group B at all time points between 2 and 8 weeks following cell transplantation (Fig. 6G) (F = 135.450, *p* < 0.05). ERG responses (b-wave and a-wave) were significantly higher in Group D than in Group C, 2 weeks following cell transplantation (*p* < 0.05). The treatment benefit can last for 8 weeks following transplantation, as evidenced by the significantly higher b-wave ERG responses in treated eyes than in control eyes.

## Discussion

In this study, we discovered that intravitreal transplantation of NSCs in DR rats resulted in long-term preservation of retinal function and significantly delayed the progression of DR for up to 8 weeks.

RGCs are the earliest-damaged cells with the highest rate of cell death, and photoreceptor apoptosis is significantly increased with the progression of DR. There are no effective measures to promote nerve repair after DR nerve damage, and exogenous cell replacement therapy may be a potential treatment for the damage^9^. NSCs have become a promising source of stem cell-based regenerative therapy. Xia *et al.* reported that MSC transplantation improved diabetic neuropathy via direct peripheral nerve angiogenesis, neurotrophic effects, and restoration of myelination^10^. In the present study, NSCs displayed trophic effects similar to those of neurons, which suggests that NSCs are a viable alternative to endogenous neurons and may be used in cell-based therapy for nerve injuries in DR^11^.

It was previously demonstrated that stem cells have protective effects against retinal vasculopathy by preventing capillary loss and retinal capillary dropout in an STZ-induced rodent model of DR^12^. These cells are particularly important in the formation of physiological vessels and improving the integrity of the blood–retina barrier^13^. It has also been demonstrated that BMSCs can increase the retinal and intravitreal concentrations of neuroprotective growth factors and provide neurotrophic effects^14^. Consistent with previous observations, intravitreal injection of stem cells results in cell clumps in the vitreous owing to the enveloping membrane of the vitreous and its high viscosity^15^. However, the molecules and trophic factors can spread on the surface of the retina and diffuse through the vitreous body^16^. We have previously demonstrated that transplanting MSCs in the vitreous can have a therapeutic effect^17^. According to previous studies, NSCs can be identified in the vitreous cavity for at least 6 weeks after intravitreal transplantation^18^. Furthermore, the cell distribution in a relatively thin cell layer could potentially increase the host-graft interaction.

Endothelial injury has been reported to occur at 4 weeks after administration of STZ^19^. The injury facilitates the infiltration of neutrophils and monocytes to the vascular wall and reduces the expression of BDNF and Thy-1, a change that propagates the initial injury to RGCs^20^. It also up-regulates retinal blood vessel leakage, which can weaken the vascular wall by disrupting the subendothelial matrix and destabilizing the medial smooth muscle cell layer^21^. Consistent with previous observations, we have demonstrated that NSCs significantly increase the expression of BDNF and Thy-1 in healthy and diabetic rats. We also found that NSCs reduce retinal blood vessel leakage, which suggests that NSCs have the ability to regulate retinal pericytes and endothelial cells and may improve the microcirculation in DR. ERG recordings demonstrated a similar therapeutic effect that lasted for 8 weeks after cell transplantation, regardless of the delivery method. These findings suggested that NSCs could increase the number of surviving RGCs in diabetic retina and improve neuroprotection to slow down the progression of DR for several weeks.

BDNF plays a major role in diabetic neuroprotection. It was shown that serum and retinal levels of BDNF are significantly reduced at the early stages of diabetes in rats^22^, and BDNF protects retinal neurons from hyperglycemia *in vitro* ^23^. In our study, we found that BDNF expression in the retina decreased in the diabetic group compared with the control group, whereas transplanted NSCs prevented the effect of experimental diabetes on BDNF expression. BDNF secreted by transplanted MSCs could reach the inner and outer nuclear layers and increase neurotrophic levels in RGCs from diabetic animals^24^. Our findings suggest that ERG preservation in diabetic animals with transplanted NSCs could involve a BDNF-dependent mechanism. However, it remains to be investigated whether NSCs directly regulate this differential expression of molecules critical for vascular remodeling. One potential mechanism is that NSCs secrete several trophic factors at the site of injury to correct this aberrant pattern of expression of vascular modeling molecules. Further studies blocking BDNF using BDNF knockout mice and the BDNF inhibitor K252a will most likely confirm that impaired vascular and neural repair in the diabetic retina involve a BDNF-dependent mechanism.

In conclusion, we demonstrated that NSCs originating from MSCs have a neuroprotective effect by increasing the number of surviving RGCs and significantly reducing the progression of DR. These results suggest that transplantation of NSCs could be a novel strategy for the treatment of neurodegeneration in DR. In future studies, we will examine the safety of this procedure in large animal models that closely mimic the human eye.

## Materials and Methods

### Cell culture

The study was approved by the Tianjin Medical University Medical Ethics Committee and complied with the Declaration of Helsinki, including current revisions, and with the Good Clinical Practice guidelines. The procedures were performed according to institutional guidelines. All subjects provided their written informed consent for human umbilical cord sampling according to the Declaration of Helsinki. We isolated and differentiated UC-MSCs according to the protocol previously published by our group^8^. Briefly, UC-MSCs: After washing with PBS and cutting into small pieces, human umbilical cords were digested with collagenase II for 1 h, followed by trypsinization for 20 minutes at 37 °C. Dissociated cells were collected and cultured in Dulbecco’s modified Eagle’s medium (DMEM)/F12 containing 10% fetal bovine serum, 100 U/ml penicillin, and 100 μg/ml streptomycin at 37 °C in a humidified atmosphere with 5% CO_2_. UC-MSCs were tested for CD73, CD90, CD105, CD34, CD45, CD11b, CD19, and HLA-D expression using flow cytometry.

For neural differentiation, the UC-MSCs were incubated for up to 3 weeks in neural differentiation medium containing 100 ng/mL neurobasal media (Santa Cruz Biotechnology, Santa Cruz, CA, USA), 50 ng/mL EGF (Sigma-Aldrich, St, Louis, MO, USA), and 2% N2/B27 (N2 and B27 Supplement, Abcam, Cambridge, MA, USA), 5% FBS, and 1% antibiotics/antimycotics. Proteomic analysis was performed to identify surface markers of NSC.

### Model preparation

Male 12-week Sprague Dawley (SD) rats were purchased from the Military Medical Academy of China. For induction of diabetes mellitus type 1, the rats were anesthetized with ketamine/xylocaine and injected with a single dose of streptozotocin (STZ) into the tail vein (50 mg/kg body weight in 5 mM pH 4.5 citrate buffer). The animals were allowed to recover for 4 days before initiation of the feeding regimen; diabetes mellitus type 1 was confirmed by measuring the levels of blood glucose with an Accu-check Sensor analyzer (Roche, Mannheim, Germany). Blood glucose was monitored throughout the study to ensure that hyperglycemia was maintained and to treat any extreme glycemic levels with insulin. Of the STZ-treated rats, only animals with blood glucose levels exceeding 300 mg/dL were considered hyperglycemic and included in the study. They were housed in separate cages under periods of 12 h of light and 12 h of dark. The rats had free access to standard chow and water. All animal care procedures were performed according to the institutional and National Institute of Health guidelines.

The rats were randomly divided into four groups: Group A (n = 42), control group without treatment; Group B (n = 42), diabetic retinopathy (DR) group; Group C (n = 42), transplanted with MSCs after DR induction; Group D (n = 42), transplanted with NSCs after DR induction. MSCs and NSCs were transplanted into the vitreous body of the rats (n = 42) at 4 weeks post-injection of STZ, an age preceding the major onset of DR. The rats were under xylocaine (10 mg/kg) and ketamine (75 mg/kg) intraperitoneal anesthesia. Cell transplantation was performed under a surgical microscope. Next, 0.2 × 10^6^ cells in 2 μL were transplanted intravitreally with a disposable insulin syringe of 0.1 mL volume, 0.30 mm (30G) × 8 mm (BD Micro-Fine Plus, Becton Dickinson). The pupils were dilated with tropicamide 0.5% and phenylephrine HCl diluted to 2%. The syringe needle was introduced with its opening facing the vitreous and close to the retina. Morphological changes in the retina were observed at 2, 4, and 8 weeks after transplantation.

### Histologic analysis

At 4 and 8 weeks after transplantation, four rats from each group were sacrificed at each time point, and their eyes were immediately removed and immersed in a cold fixative of 4% glutaraldehyde in 0.1 M phosphate buffer. The corneas were removed, and the eyes were left in the fixative for 24 h. The lenses were then removed, followed by dehydration with a graded series of increasing ethanol concentrations, and the eyecups were then embedded in paraffin. The paraffin-embedded sections were stained using routine hematoxylin-eosin (HE) staining, and each section was examined by blinded observers. Briefly, retinas were oxidized with 1% periodic acid and treated with Schiff reagent. The number of cells in the retinal ganglion cell (RGC) layer within 10 demarcated regions of one single slide was recorded randomly, and the average number of cells in the RGC layer was quantified and recorded from three serial slides.

### Measurement of blood–retinal barrier (BRB) breakdown using Evans Blue (EB) dye

At 8 weeks after transplantation, six rats from each group were deeply anesthetized, and EB dye (30 mg/mL in saline; Sigma) was injected through the tail vein over 10 s at a dosage of 45 mg/kg. Retinal vascular permeability was quantified according to the procedure of Qaum *et al.* ^25^. BRB breakdown was calculated as previously described, and the values were expressed as EB (ng) × retinal weight (mg^−1^).

### Immunohistochemistry

At 4 and 8 weeks after transplantation, four rats from each group were sacrificed at each time point. The eyes were removed and fixed in 10% formaldehyde solution. Serial 4-μm paraffin retinal sections were stained with polyclonal rabbit anti-rat brain-derived neurotrophic factor (BDNF) and Thy-1 antibody (Boster Biological Technology, Wuhan, China). Negative controls were obtained by replacing the primary antibody with PBS. The staining was visualized by a reaction with 3,3-diaminobenzidine tetrahydrochloride (DAB; Sigma Chemical Co., USA). Random sections (6 per retina) were imaged using light microscopy at 200x magnification. Immunostaining for BDNF and Thy-1 was quantified using a computer imaging analysis system and particle counting algorithm standardized across all 4 groups according to the procedure of Chang CH *et al.* ^26^.

### Western Blotting Analysis

At 8 weeks after transplantation, six rats from each group were deeply anesthetized. Western blotting analyses were performed using standard methods. Briefly, retinas were carefully dissected from the rat eyes. The protein concentration in the tissue lysates was measured by protein assay (Bradford Protein Assay; Bio-Rad, Hercules, CA, USA). Proteins were transferred electrophoretically onto polyvinylidene fluoride (PVDF) membranes (Millipore, Billerica, MA, USA), and the membranes were blocked with 5% skim milk and then incubated with BDNF (1:1000 in 5% BSA) or β -actin (1:5000 in 5% BSA) (ZSGB-BIO, Beijing, China) primary antibodies at 4 °C overnight. Next, the membranes were incubated with HRP-conjugated secondary antibodies (Santa Cruz, CA, USA) at room temperature for 2 h and visualized using the ChemiDoc™ MP System (Bio-Rad, Hercules, CA, USA). The protein bands were quantified using ImageJ software for statistical analysis.

### Electroretinogram (ERG) recording

At 2, 4, and 8 weeks after transplantation, four rats from each group were deeply anesthetized at each time point. For dark-adapted ERG, the rats were kept in total darkness for 12 h for testing. The animals were deeply anesthetized, and the pupils were dilated with topical 1% tropicamide. ERGs were recorded from both eyes simultaneously using golden wire loops on the corneas. A chloride silver reference electrode was placed subcutaneously near the temporal canthus. The ground electrode was placed on the tail. For dark-adapted ERG, responses were averaged with stimulus intervals of 1 to 30 s depending on the stimulus light intensity. For light-adapted ERG, the animals were light-adapted for 10 min prior to testing, and the responses were averaged with stimulus intervals of 1 s.

### Statistical analysis

The data were presented as the mean ± SEM, and the data were compared using one-way analysis of variance (ANOVA). A *p*-value < 0.05 was considered statistically significant. All analyses were performed using a statistical software package (SPSS 13.0, Chicago, IL, USA).
